# Reflections on Gender Analyses of Bibliographic Corpora

**DOI:** 10.3389/fdata.2019.00029

**Published:** 2019-08-28

**Authors:** Helena Mihaljević, Marco Tullney, Lucía Santamaría, Christian Steinfeldt

**Affiliations:** ^1^Hochschule für Technik und Wirtschaft Berlin, University of Applied Sciences, Berlin, Germany; ^2^Technische Informationsbibliothek (TIB), Hanover, Germany; ^3^Amazon Development Center, Berlin, Germany

**Keywords:** gender, reproducibility, data science, bias, societal issues, science studies, automatic gender recognition

## Abstract

The interplay between an academic's gender and their scholarly output is a riveting topic at the intersection of scientometrics, data science, gender studies, and sociology. Its effects can be studied to analyze the role of gender in research productivity, tenure and promotion standards, collaboration and networks, or scientific impact, among others. The typical methodology in this field of research is based on a number of assumptions that are customarily not discussed in detail in the relevant literature, but undoubtedly merit a critical examination. Presumably the most confronting aspect is the categorization of gender. An author's gender is typically inferred from their name, further reduced to a binary feature by an algorithmic procedure. This and subsequent data processing steps introduce biases whose effects are hard to estimate. In this report we describe said problems and discuss the reception and interplay of this line of research within the field. We also outline the effect of obstacles, such as non-availability of data and code for transparent communication. Building on our research on gender effects on scientific publications, we challenge the prevailing methodology in the field and offer a critical reflection on some of its flaws and pitfalls. Our observations are meant to open up the discussion around the need and feasibility of more elaborated approaches to tackle gender in conjunction with analyses of bibliographic sources.

## 1. Introduction

Despite the increasing number of women entering the Science, Technology, Engineering and Mathematics (STEM) fields, gender inequities persist. Women leave academia at a higher rate than their male colleagues, leading to significant female underrepresentation, particularly in permanent academic positions. A successful academic career has long been inextricably tied with a prolific scholarly record; scientific publications are not only the major outlet for scholarly communication, they are regarded as a proxy for a researcher's scientific credo and are one of the key factors in achieving and maintaining a flourishing career in academia. A natural question arises whether women and men differ in their publication practices in a way that contributes to the observed gender gap in STEM.

With the digitization of bibliographic metadata it became possible to approach this matter on a large scale using algorithmic, statistical, and computational methods. Several studies have leveraged existing databases to investigate the role of gender in academic publishing, either with a general focus (Larivière et al., 2013; West et al., 2013) or for particular disciplines, such as mathematics (Mihaljević-Brandt et al., 2016) or biology (Bonham and Stefan, 2017). In Mihaljević-Brandt et al. (2016), we analyzed the scholarly output of about 150,000 mathematicians who authored over 2 million research articles since 1970. We showed that women abandon academia at a larger rate then their male counterparts, at different stages of their careers. We focused on aspects known to have a strong impact on career development, and concluded that, on average, women mathematicians publish in less prestigious journals and appear less frequently as single authors while they collaborate with a comparable-sized network of peers. These results prompted the interest for extending this line of analysis to other disciplines, work that is being continued in an ongoing interdisciplinary project^1^.

Within the course of our investigations we have faced a number of critical aspects that are worth examining more closely. While we are certain that our results are relevant and reliable, we believe that some of the underlying assumptions and methods, though deemed valid and adequate given the available resources, deserve to be examined in more detail. Our ultimate goal is to foster a discussion on critical and sensitive topics that may potentially be encountered when making statements about individuals and existing societal issues based on publication metadata.

In this article we review a series of concerns that arise after critical examination of the core assumptions that ordinarily underlie gender inference from bibliographic data sources. We inspect common biases induced by gender assignment algorithms and other common data processing steps applied to bibliographic records. Finally, we discuss the reception and interplay of this kind of research within the field, and reflect on the issue of data and code availability and its effect on scientific standards like reproducibility. We discuss potential alternatives in order to foster a debate about best practices for subsequent projects.

## 2. Critical Aspects of the Analysis of Gender in Scholarly Publications

### 2.1. Assessing Humans

In bibliometric studies, the author's name is often the only piece of information susceptible of providing an indication of their gender. Name-to-gender inference is typically performed using a combination of multiple steps that usually involve querying name repositories like censuses or birth lists as well as applying insights from sociolinguistics. This is precisely how we approached the gender inference task in Mihaljević-Brandt et al. (2016). Recent analogous studies are increasingly making use of web services that continuously gather data from multiple sources. The results are sometimes augmented by applying, e.g., face recognition software to images retrieved when using a search engine to look up the author's name string.

Many issues arise in connection with said approaches. The resulting processes are seldom transparent, reproducible, or transferable; most studies relying on name-based gender inference fall short on thoroughly evaluating potential biases (Santamaría and Mihaljević, 2018). Enhancing name-based gender inference by facial analysis algorithms might incur an additional significant bias, particularly against darker-skinned women (Buolamwini and Gebru, 2018). Moreover, such approaches only allow for a binary definition of gender, which fundamentally excludes individuals that do not conform to this societal concept. This topic is typically not further discussed in the relevant literature. Ultimately and from a statistical point of view, this exclusion is considered “bearable”: the estimated share of transgender and other non-binary authors is considered low enough that the binary gender simplification does not significantly distort the results. And yet, this enormously diminishes the needs and practices of transgender authors. Moreover, from the perspective of an individual who identifies outside the binary model, every such study is another manifestation of a “misgendering” practice in which the person is refused to be considered as part of the target group. In fact, automatic misgendering from an algorithm tends to be perceived as even more harmful than if it originated from another person (Hamidi et al., 2018).

The problem lies in the basic idea of inferring a person's gender form an attribute, such as the name string: personal names are assigned to individuals at birth as part of a schema based on a binary, immutable, and physiologically determined definition of gender (Keyes, 2018), much like other automatic gender recognition systems based on features, such as face, body, movement, or voice (Hamidi et al., 2018). Hence any approach that automates gender recognition (AGR) through a third-party mechanism, be it algorithmically or via human judgment, denies the view that one's gender identity is subjective (Butler, 1988), and embodies an old concept: an “incongruous pairing of futuristic AGR technology with old-fashioned conceptualizations of gender and its value to society” (Hamidi et al., 2018, 7), or as D'Ignazio (2016) puts it: “Non-binary genders will always be outliers.”

Gender-inclusive bibliometric analyses can become possible only when no names or photographs are used as proxies for gender, allowing authors to define their gender autonomously instead. We have frequently thought about different approaches toward self-identification. A first idea was to draw a sample of authors and ask them to volunteer their gender. The drawbacks quickly become apparent, since authors can only be contacted via information taken from the publication's metadata. This introduces several issues: not every author provides their e-mail addresses, as often only the lab's or research group's PI is listed as corresponding author; then, only part of the contacted researchers would respond to such a request, which further prevents the creation of a random subsample; finally, the legal ramifications of using e-mail addresses for this purpose are far from clear. Moreover, the procedure would have to be repeated for every new study, leading to an unfeasible approach. Especially the latter argument begs for a sustainable and scalable solution. A second idea was to provide a web service to facilitate gender self-identification. If taken seriously, such an infrastructure should not be part of a time-limited research project, but instead exist as a persistent service, preferably run by a suitable organization. Such a service would presumably take a long time to become widespread in the scientific community, even if researchers considered it meaningful enough to provide data.

It is therefore impossible to accurately assign a gender to all authors without misgendering certain groups of individuals, and it seems difficult to design and implement a service for self-identification to generate a solid database that could be utilized for sound statistical analyses. This begs the question of whether such analyses are in fact necessary and what benefit they provide to societal development. Every analysis bears the risk of reinforcing gender stereotypes and binary gender models. External attribution of properties like gender is not only difficult and biased, it is an infringement of the autonomy of the people who are subjected to it: “Simply starting with the assumption that all data are people until proven otherwise places the difficulty of disassociating data from specific individuals front and center” (Zook et al., 2017). There should be a good reason to conduct analyses that require assigning gender to individuals; we decided to perform them because academia is notoriously not gender-agnostic and because gender differences can be observed and need to be explained. Yet there is a fine line between analysing gender inequalities and reinforcing gender as a category, and we still would like to see processes like publishing and hiring become as gender-agnostic as possible.

### 2.2. Simplification and Selection Biases

The preparation of bibliographic records involves various algorithmic routines, which might be rule-based (e.g., comparison of affiliation strings with geo-databases), rely completely on third-party sources (e.g., usage of name-to-gender probabilistic assignments from commercial web services), or involve non-trivial machine learning models (e.g., linkage of authorship records to author entities). Thus, the resulting data set is the product of multiple data preprocessing steps and as such naturally susceptible to errors. It is best practice to estimate the inaccuracies of the involved procedures as realistically as possible, in particular when modeling social phenomena. However, this is often a highly complex and resource-consuming task that unsurprisingly falls short on many occasions, not only in commercial data science projects but also in scientific studies.

Large data sets typically require more preprocessing work. On the positive side, and in contrast to empirical work based on small samples, researchers can afford to exclude data points that do not contain sufficient information for the subsequent data mining steps (or, in other words, contain missing values in relevant variables that cannot be adequately inferred). At the same time, removal of data points induces bias. An illustrative example is the exclusion of the majority of Chinese names: these can stem from thousands of characters whose multiple meanings frequently reflect certain gender stereotypes. Much of this information is lost through romanization, which normally takes place when Chinese authors publish in Western journals.

The example above illustrates two kinds of biases often encountered in bibliographic analyses (Ridge, 2015): selection bias, which describes the tendency to skew data sources toward the most accessible subsets, and sampling or exclusion bias, which introduces a distortion of the data sets toward certain subgroups. Analogous examples abound: record linkage algorithms work worse for authors with very common names; author profiles of women are more often incomplete due to larger probability of family name changes; researchers with names of East-European origin are harder to cluster due to varying spellings from different name transliterations. This list is far from complete but already indicates that a precise specification and quantification of the biases induced through preprocessing is practically impossible.

While bias is typical for projects and applications from data science or machine learning, it is regularly left unaddressed in many business applications and scientific projects. This is somewhat surprising given the fact that data science practitioners often have a background in traditional sciences, where the identification and removal of bias when reasoning about the world are of high importance (Ridge, 2015). Luckily, there is a growing number of research communities, such as “Fairness, Accountability, and Transparency in Machine Learning” (FATML) that address the transparency of algorithmic decisions and the reduction of induced biases, partly in reaction to recent examples of discrimination caused especially by computer vision software amplifying existing societal prejudices.

Recommendations on how to recognize and avoid bias in data science are increasingly becoming mandatory, leading to the formulation of judicious best practices that ought to be implemented regardless of the concrete task at hand. In order to make research as transparent and reproducible as possible, one should at the very least track raw data sources comprehensively; provide quantitative and qualitative information about them; record and summarize data processing pipelines; describe all data transformations and explore their effect; and write and publish reproducible code (Ridge, 2015). Recent work by Gebru et al. (2018) formalizes this in a sense by proposing a framework to document data sets with data sheets containing a list of standardized questions: why a data set was created, who funded it, what preprocessing has been done, and in case it relates to real people, whether they agreed to the data usage. Still, these best practices will be challenged in many projects, especially in those that make use of closed data not available for secondary analyses.

### 2.3. Interaction With the Field

An intriguing and partly surprising result in Mihaljević-Brandt et al. (2016) is the underrepresentation of female authors in high-ranked journals, evaluated with respect to two prominent ranking schemes. In mathematics, as well as in other fields, it is commonsensical to expect the perceived quality of the journals where authors publish to be relevant for their scientific career. However, we cannot quantify how relevant it is. The available data does not allow us to transfer our found correlation between gender and journal rank into a model for the observed gender gap in mathematics. Modeling female mathematicians' careers would require much more information beyond publication data, thus no inference or predictive model can be produced based solely on studying bibliometric records.

Yet in fact, we are certain that the observed inequality regarding top-journal publications is causally related to the higher drop out of women mathematicians, but we cannot prove it. A causal link seems probable, but has not been found: “An interesting pattern, by definition, is one that has a non-negligible subjective or logical probability of being potentially explicable, at least in part. It is possible to judge that a pattern has an underlying explanation even if we are unable to find it” (Good, 1983). The proof of a causal effect usually requires some sort of experiment, but the most one can really expect from working with observational data is correlation. As argued further in Villa (2018), there are still certain benefits of talking about causality explicitly even if it may not be demonstrable. For one thing, we constantly operate like this without being able to perform confirmatory experiments, but, more importantly, it suits the purpose of the undertaken data analysis: “When you analyze data [it] is because you want [to] arrive to some conclusions to take further actions. If you think in that way, is because you think those actions affect (and thus are a cause of) some quantity of interest. So, even [when] you talk about correlations for technical correctness, you are going to use those insights in a causal way” (Villa, 2018).

Although we are able to exclude the choice of subfield as a relevant factor, we cannot conclusively deduce why women publish less in high-ranked journals. Are women simply less likely to submit an article to them, or are they more frequently rejected? To fill the “causality gap” we resorted to a different data source. We recently conducted a global survey of scientists in STEM, in which participants were also asked to quantify the number of their publications submitted to a renowned journal within the last 5 years. A preliminary evaluation of the responses indicates that, on a global scale, women and men perceive that their submission practices in that respect are comparable.

Considered as part of the big picture, our result is thus a good example of what Tukey (1962) calls “approximate knowledge,” referring to the maxim that data analysis progresses by offering approximate answers to the right questions. It also showcases the importance of exploratory analyses, which are essential to be able to formulate appropriate discussion points and to plan further data acquisition (Tukey, 1993). Presently it seems sensible to demand more transparency from publishers regarding their publication acceptance data. Journal rejection rates split by gender should be openly shared, since that would ultimately help elucidate the reasons for the underrepresentation of women in “renowned” journals. The formulation of such demands, though, would position one's own work within a system of institutional decision-making, moving it further away from a descriptive approach which rather focuses on revealing differences between genders within academia. While a descriptive approach might appear more “objective” and pure, it is arguable whether bibliometric research can be isolated in that way at all. As discussed in Angermuller and van Leeuwen (2019), who studied the societal role of bibliometric and scientometric research from Michel Foucault's perspective on science as power-knowledge, descriptive research that uses numbers to represent social realities is necessarily a constitutional part of such realities. As such, bibliometric research “cannot simply render a given state of the social world reality without intervening in it.”

Certainly, our research can be used to compare groups of individuals, and it is challenging to estimate the exact effect it might have on academic decision-making. For instance, the conclusion that women publish less than men in a given period of time can be used to justify the lack of women among professors or grant recipients. Thus, without placing results within the right context and formulating clear goals, research on effects of gender on publication practices could help objectify and justify already existing inequalities between groups of academics. We believe, however, that this demands domain-specific expertise, which is crucial to be able to formulate relevant research questions for different fields or “to balance appropriate assumptions with computationally efficient methods” (Blei and Smyth, 2017). As posed in Good (1983), “even an exploratory data analyst cannot expect to obtain truly deep results in a science with which he is unfamiliar unless he cooperates with a scientific specialist.”

One other obstacle when communicating results of data-driven research is the non-availability of data, code, and other artifacts that would enable reproducibility of the findings, identification of errors, or creation of derived investigations. Making research openly available includes providing open data and openly published software code. This is especially important if working on big data sets when far-reaching preprocessing steps are applied. In fact, reproducibility is one of the key requirements of (at least) future research (Donoho, 2017) (less critical are Shiffrin et al., 2017). Many data sources are not open. In our research we used paywalled databases, especially the large zbMATH corpus. We archived data and code and ensured that it can be accessed—if the rightholders of the database allow. This is not optimal, yet it is a first step. But in a general sense and for a broader public, our research is not reproducible—as it is the case of many data science projects.

When research results shall influence people's lives, every necessary step should be taken to make studies as reliable as possible. Data needed to reproduce the findings has to be archived, and its long-term availability ought to be guaranteed (Waltman et al., 2018). When working with open data, a data repository has to be found. When working with closed data, additional steps are necessary to ensure that other researchers will be able to access it. Relying on data not available for secondary analyses should be the very last resort, and researchers shall always try to make their data and software accessible. This might include negotiating with rightholders of databases. These efforts should at least be documented, if working with non-open data and code seems inevitable in some cases. At the very least it should be possible for other researchers to have a way to check the original results. A special meaning comes to this question when we talk about bias in research designs, data and algorithms. A middle ground that could be used more is the provision of aggregated data and visualizations, including interactive ones that offer researchers and other interested parties a better insight into the data and findings (we are following this path in our current project).

## 3. Discussion

With each publication of their research findings, scientists expose their work to the public. But scientists themselves might become data points for measurements or analyses of scientific practices, often without being aware of the concrete usage of their data and without the possibility to interact or exert any influence on it. This is in particular the case when demographical features, such as gender or country of origin, are the subject of investigations. It is thus of the utmost importance for data scientists working in this field to “recognize the human participants and complex systems contained within their data and make grappling with ethical questions part of their standard workflow” (Zook et al., 2017).

We have discussed some troublesome but fundamental aspects frequently encountered in analyses of bibliographic records with respect to gender. We have problematized the process of inferring an author's gender solely from metadata like a name string, which is not only in stark contrast with a subjective and internal perception of gender but also runs the risk of misgendering individuals who do not conform to the gender binary. Due to a lack of alternatives that do not infringe the subject's autonomy, and the risk of reinforcing gender stereotypes and binary gender models, we find it important to keep questioning the necessity of any given gender-related data analysis and to compare the objectives and effects of our own research (to disclose gender inequalities) with the methodological compromises we make (e.g., reinforcing a binary gender model). For research like ours that lies at the intersection of data science and sociology, it is paramount to reflect on the interpretations and usages of one's research within the field. We believe that it is almost impossible to treat such research as solely descriptive or exploratory; we would instead propose considering the research context more closely and formulating the goals in a transparent way in order to minimize the risk of misusage for objectification or reinforcement of existing inequalities. In our opinion, a solid contextualization of analyses involving social phenomena and human participants demands domain-specific expertise, ultimately leading to interdisciplinary collaborations. Such collaborations, especially those involving qualitative methods, might be able to shed some light on the mechanisms that lead to the observed differences between male and female authors.

In Mihaljević-Brandt et al. (2016), we highly benefited from our expertise in mathematics and gender studies, in data science and in working with bibliometric data. We believe that previous domain knowledge helps to address shortcomings, such as the recognition of biases induced through data selection and processing and their potential effects. This topic, while often neglected in studies based on exploratory data analyses, is of high relevance for the actual conclusions that follow from the obtained results. The difficulty of specifying and quantifying the bias more precisely, but also the natural demand for reproducibility of research, make it all the more important to provide open access to raw data plus the software code. The analysis of bibliographic data is often based on closed data sources stored in paywalled corpora. Since such research has the potential to influence people's lives, we believe that scientists in this field should put considerable efforts into finding acceptable solutions and compromises with the rightholders of databases.

These hurdles are not easy to overcome. Domain expertise can be ensured by inviting researchers from the field to collaborate, thus fostering multidisciplinary research. This, however, might lead to difficulties, e.g., due to mainstream expectations in a discipline. Given the ubiquity of commercial bibliographic databases, ensuring sustainable access to comprehensive open bibliographic data will need additional and combined efforts of researchers and others (e.g., librarians).

## Author Contributions

HM and MT conceived the idea for the report and wrote the first draft of the manuscript. CS and LS contributed to the design and to specific sections. HM, LS, and MT edited and corrected the text. All authors read and approved the submitted version.

### Conflict of Interest Statement

LS was employed by company Amazon. The remaining authors declare that the research was conducted in the absence of any commercial or financial relationships that could be construed as a potential conflict of interest.

## Footnotes

^1^“A Global Approach to the Gender Gap in Mathematical, Computing, and Natural Sciences: How to Measure It, How to Reduce It?” https://gender-gap-in-science.org
